# Management of Thyroid Cancer and Related Nodular Disease

**DOI:** 10.1038/sj.bjc.6603397

**Published:** 2006-10-31

**Authors:** A Cassoni

**Affiliations:** 1 University College Hospital, London, UK

Apart from the chapter on pathology, this is a single author book and has most of the advantages and few of the drawbacks of such an approach. The author is a nuclear medicine physician/‘thyroidologist’ with several decades of recent practice at Stanford in USA, preceded by working in the UK and enriched by sabbaticals elsewhere. The target audience is wide, mostly within the medical specialties in a wide range of disciplines but also nurses, students and patients. The detail of the text and comprehensive scope of the reviews make it entirely suitable for those with a major interest in thyroid cancer who will appreciate it for its clear presentation of principles and rationale for treatments and as a guide to the management of difficult problems.

An understanding of basic principles is not assumed. The sections on epidemiology and radiation physics and dosimetry especially develop the principles clearly and logically and are well worth attention. It therefore functions well both as a book to dip into for specific questions and as a comprehensive presentation of the evidence and process necessary to provide a modern practice. There is a consistent approach with a logical progression between the chapters that reinforce the principles without undue repetition. The broad personal experience provides a series of anecdotes and, while personal preferences are certainly expressed, these are always placed on an extensive and balanced background of literature review.

The volume is some 398 A4 pages long and comprises 12 chapters. There are extensive references for each ranging from 60 or so for the epidemiology to 680 for the main management chapter. The majority relate to papillary and follicular tumours but lymphoma, medullary and anaplastic cancers are presented. The anatomy, physiology, aetiology and pathology are well covered with up to date information, especially on the genetics. There is good use of illustrative photographs and clear explanatory diagrams. The principles of nuclear medicine scanning, the interpretation of the scans and the physics underlying therapy are explained from first principles and would be very valuable to all non-nuclear medicine physicians in the field.

He does not fail to tackle the current controversial issues that are the extent of surgery necessary, the need for ablation, the use and difficulties with thyroglobulin and the developing role of recombinant TSH. Very specific guidelines are outlined and examples of information sheets provided. These could be easily adapted for centres needing to update their own written information.

I have few and minor reservations. In view of the detail in which the majority of the book is presented, a greater discussion about loco-regional relapse, its impact on the patient and its management and outcome would have been a welcome addition. It is generally very well presented but there are places where the figures are at some distance from the text that refers to them and impedes the ease of reading.

In summary this is a well-presented text, comprehensive in content, logical and well presented. It combines clear management principles based on both personal experience and extensive familiarity with the literature. It is highly recommended for those with a major interest in thyroid cancer and would be a good reference volume on this topic for a library.

